# Multi-Objective Optimization of Tool Edge Geometry for Enhanced Cutting Performance in Turning Ti6Al4V

**DOI:** 10.3390/ma18174160

**Published:** 2025-09-04

**Authors:** Zichuan Zou, Ting Zhang, Lin He

**Affiliations:** 1 School of Mechanical & Electrical Engineering, Guizhou Normal University, Guiyang 550025, China; 2 School of Management, Guizhou University, Guiyang 550025, China; ttkx327@163.com; 3 School of Mechanical Engineering, Guizhou University, Guiyang 550025, China; helin6568@163.com

**Keywords:** cutting tool, co-simulation, multi-objective optimization, micro-groove structure, titanium

## Abstract

Tool structure design methodologies predominantly rely on trial-and-error approaches or single-objective optimization but fail to achieve coordinated enhancement of multiple performance metrics while lacking thorough investigation into complex cutting coupling mechanisms. This study proposes a multi-objective optimization framework integrating joint simulation approaches. First, a finite element model for orthogonal turning was developed, incorporating the hyperbolic tangent (TANH) constitutive model and variable coefficient friction model. The cutting performance of four micro-groove configurations is comparatively analyzed. Subsequently, parametric modeling coupled with simulation–data interaction enables multi-objective optimization targeting minimized cutting force, reduced cutting temperature, and decreased wear rate. The Non-dominated Sorting Genetic Algorithm II (NSGA-II) explores Pareto-optimized solutions for arc micro-groove geometric parameters. Finally, optimized tools manufactured via powder metallurgy undergo experimental validation. The results demonstrate that the optimized tool achieves significant improvements: a 19.3% reduction in cutting force, a 14.2% decrease in cutting temperature, and tool life extended by 33.3% compared to baseline tools. Enhanced chip control is evidenced by an 11.4% reduction in chip curl radius, accompanied by diminished oxidation/adhesive wear and superior surface finish. This multi-objective optimization methodology effectively overcomes the constraints of conventional single-parameter optimization, substantially improving comprehensive tool performance while establishing a reference paradigm for cutting tool design under complex operational conditions.

## 1. Introduction

Metal cutting is a pivotal manufacturing process in the equipment industry, with its technological sophistication directly governing the operational reliability and production efficiency of high-end machinery. Driven by escalating demands for high-performance components in the aerospace, defense, and military sectors, the utilization of challenging materials like Ti6Al4V has risen markedly. As a typical dual-phase alloy, Ti6Al4V exhibits poor machinability. During cutting, the material exhibits a significant thermomechanical damage coupling effect, where high-strain-rate plastic deformation induces localized adiabatic shear phenomena, ultimately compromising surface integrity and tool life. Achieving precise control of material separation through innovative tool edge design has thus become a critical challenge in overcoming bottlenecks in advanced equipment manufacturing.

The cutting-edge profile, defined as the transitional zone between a tool’s rake face and flank face, critically governs material flow dynamics and chip formation through its geometric configuration. This influence primarily manifests in two key aspects: the fundamental chip formation mechanism and the minimum achievable cutting thickness [1]. During material removal, edge geometry dictates chip generation by regulating the nucleation and propagation trajectories of shear slip bands [2]. Specifically, negative rake edges facilitate shear-dominant material separation, whereas positive rake edges preferentially trigger crack-dominant separation [3]. An enlarged edge hone radius intensifies the plowing effect, inducing the nonlinear growth of the minimum cutting thickness through plastic material accumulation beneath the tool [4]. Recent advancements in tool design have prioritized surface texturing on rake faces. Engineered micro-textured structures enhance machining performance by reducing cutting temperatures and forces, thereby prolonging tool lifespan through mitigated wear and suppressed workpiece material adhesion. These structures also modulate interfacial properties including friction coefficients, wettability, and adhesion characteristics, collectively contributing to eco-efficient manufacturing practices [5]. Current micro-texturing techniques, however, remain constrained to simple geometries (e.g., linear grooves or dimple arrays) due to fabrication limitations [6]. Emerging technologies like laser-assisted machining and powder metallurgy now enable complex edge geometries. Beyond traditional linear and hybrid linear-arc grooves, novel asymmetric profiles, and three-dimensional curved grooves are being developed to address specialized machining challenges [7]. Despite the widespread adoption of micro-textured tools for their superior chip fragmentation, curling, and evacuation capabilities, existing designs predominantly rely on empirical parameter optimization targeting isolated performance metrics. This methodology inadequately accounts for the intricate thermomechanical coupling effects inherent to cutting processes and lacks mechanistic insights into tool–workpiece interfacial interactions, potentially limiting optimization efficacy.

Contemporary studies on tool structure optimization predominantly adopt single-objective frameworks targeting isolated machining metrics—cutting force reduction, thermal management, or surface quality enhancement—while employing trial-and-error iterations or orthogonal experiments to identify optimal parameters within constrained ranges. Deng [8] developed a wear-aware cutting-edge design methodology, systematically evaluating the influence of edge hone radius, clearance angle, and wedge angle on thermal gradients and stress fields. Cascón [9] optimized chip-breaking groove geometries via orthogonal experimentation, designing a novel groove profile prioritizing chip fragmentation efficiency. Zou [10] leveraged thermal field characteristics to refine a singular asymmetric micro-groove configuration adjacent to the primary cutting edge. These parameter-tuning approaches, reliant on sequential orthogonal or single-variable testing, prove computationally intensive and suboptimal for global parameter space exploration. Emerging methodologies integrate multi-objective intelligent algorithms to balance competing performance criteria [11,12]. Chen [13] highlighted the necessity for next-generation tool design systems to holistically incorporate workpiece materials, operational conditions, and coating properties into finite element method (FEM)-based simulations. This vision necessitates developing customized software interfaces to automate FEM-guided geometric optimization within design workflows. Yue [14] pioneered an integrated geometry–performance–application design paradigm, utilizing NSGA-II to optimize tool parameters against wear resistance and material removal rate. While current research predominantly constructs surrogate models to correlate tool geometry with machining outcomes, these models suffer from high computational costs and insufficient predictive accuracy.

In conclusion, conventional tool optimization methodologies remain predominantly experience-driven and reliant on trial-and-error practices, lacking robust theoretical foundations while narrowly targeting isolated performance indicators. Even existing multi-objective structural optimization studies often reduce complexity through oversimplified weighting strategies, proving inadequate for heterogeneous material and machining scenarios. To address these limitations, this work develops a two-dimensional (2D) turning simulation framework integrating the TANH constitutive model and variable-coefficient friction model. Arc-profile micro-grooves are preliminarily screened for balanced performance characteristics and subjected to multi-objective optimization. Incorporating a constant time interval wear rate hypothesis for predictive wear modeling, the NSGA-II algorithm synergizes parametric modeling with Python-based data automation to optimize groove geometry against tripartite objectives: minimized cutting forces, reduced thermal loads, and suppressed wear rates. Prototype tools manufactured from optimized parameters undergo rigorous machining trials, experimentally validating the methodology’s reliability.

## 2. Classification and Cutting Performance Analysis of Micro-Groove Structures

### 2.1. Geometric Modeling of Micro-Groove Structures

**Figure 1:**
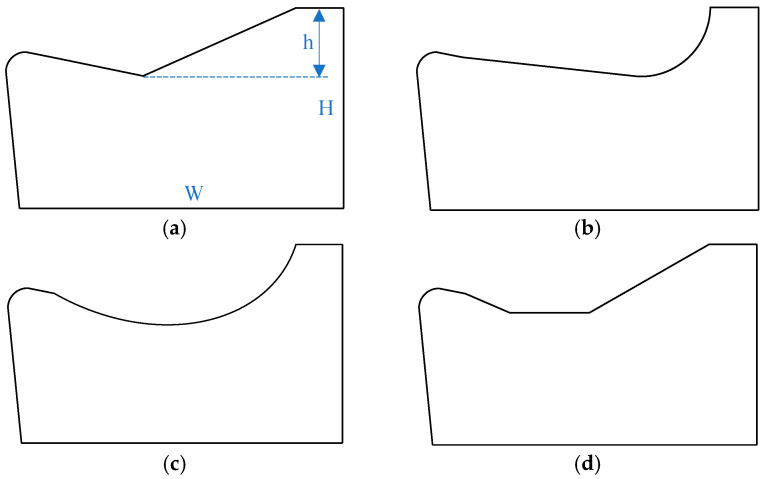
Schematic diagram of groove structure: (**a**) Straight Type; (**b**) Straight-Arc Type; (**c**) Arc Type; and (**d**) Trapezoidal Type.

Typical micro-groove configurations on tool rake faces comprise four geometries—the Straight Type, Arc Type, Straight-Arc Type, and Trapezoidal Type—as schematically depicted in Figure 1. To ensure simulation consistency, all models share uniform dimensions: height (H) = 1 mm, width (W) = 1.2 mm, clearance angle = 7°, and groove depth (h) = 0.3 mm. Straight-Type and Trapezoidal-Type grooves comprise intersecting straight sections. The forward section adjacent to the tool tip directs chip flow, while the rear section promotes progressive chip curling and fracture. The Straight-Arc Type integrates a straight front section with a rear arc segment, where the arc replaces the linear rear section of other types to regulate curling dynamics. Reducing the arc diameter amplifies curling stress, accelerating chip fracture. Arc-Type grooves feature an inherently larger rake angle than the other three types, generating smaller chip curl radii, higher deformation levels, and enhanced fracture efficiency.

### 2.2. Finite Element Model Construction

#### 2.2.1. Metal Material Properties

The workpiece material consists of Ti6Al4V bar stock (manufacturer: Shaanxi TianCheng Aerospace Company, Xianyang, China), with its chemical composition provided in Table 1 (not required for input into the simulation software and provided for reference only) and its physical and mechanical properties detailed in Table 2.

The material constitutive model governs mechanical behavior by integrating deformation parameters including strain, temperature, and strain rate. The reliability of finite element simulations critically depends on constitutive model selection. While the Johnson–Cook (J-C) constitutive model finds broad application, it inadequately characterizes the temperature and strain rate-dependent plasticity of Ti6Al4V. Calamaz [17] resolved this limitation by augmenting the J-C framework with a strain softening function, formulating the TANH constitutive model with its mathematical expression detailed below.

(1) $$\sigma =\left[A+B\varepsilon^{n}\left(\frac{1}{\exp\left(\varepsilon^{a}\right)}\right)\right]\left[1+Cln\left(\frac{\dot{\varepsilon }}{{\dot{\varepsilon }}_{0}}\right)\right]\left[1-{\left(\frac{T-T_{r}}{T_{m}-T_{r}}\right)}^{m}\right]\left[D+\left(1-D\right)\tanh\left(\frac{1}{{\left(\varepsilon +S\right)}^{c}}\right)\right]$$

(2) $$D=1-{\left(\frac{T}{T_{m}}\right)}^{d}$$

(3) $$S={\left(\frac{T}{T_{m}}\right)}^{b}$$

where ε denotes the equivalent plastic strain; $\dot{\varepsilon }\;$ represents the equivalent strain rate; ${\dot{\varepsilon }}_{0}$ is the reference strain rate, ${\dot{\varepsilon }}_{0}=1\;s^{-1}$; $T_{r}$ is the room temperature (25 °C); $T_{m}$ is the melting temperature; T denotes the deformation temperature; A represents the initial yield stress; B is the material strain strengthening coefficient; C expresses the strain rate sensitivity; n is the hardening index; and m is the thermal softening index. The variables a, b, c, and d in the equation serve as correction coefficients for the TANH constitutive model, governing its strain softening behavior. The full set of model parameters is provided in Table 3.

To address diverse damage mechanisms, ABAQUS incorporates multiple metal damage models such as Ductile Damage, J-C Damage, and Shear Damage. The J-C Damage failure model [19] enhances the Hancock–Mackenzie fracture criterion [20] by incorporating strain rate, temperature, and hydrostatic pressure dependencies into fracture strain calculations, enabling precise simulation of element deletion during material failure. Consequently, the J-C Damage model serves as the material’s primary damage criterion, defined mathematically in Equation (4)

(4) $$D=\sum \frac{\Delta \varepsilon^{pl}}{\varepsilon_{f}^{pl}}$$

The damage parameter  D, equivalent plastic strain increment $\Delta \varepsilon^{pl}$, and equivalent plastic strain $\;\varepsilon_{f}^{pl}$ are governed by the relationships defined in Equation (5) [21].

(5) $$\varepsilon_{f}^{pl}=\left[D_{1}+D_{2}\exp\left(D_{3}\sigma^{*}\right)\right]\left[1+D_{4}{ln}_{\varepsilon^{*}}\right]\left[1+D_{5}\left(\frac{T-T_{r}}{T_{m}-T_{r}}\right)\right]$$

where $\sigma^{*}$ is stress triaxiality, p is hydrostatic pressure, and $\varepsilon^{*}$ is a dimensionless strain rate; J-C damage failure parameters with values are tabulated in Table 4.

#### 2.2.2. Friction Model

In metal cutting, tribological interactions occur between the tool rake face and the chip/workpiece, as well as between the flank face and the machined surface. These frictional effects induce significant fluctuations in temperature, stress, and strain fields, critically governing plastic deformation behavior. While conventional simulations rely predominantly on static friction models (e.g., Coulomb and its modified variants), their assumption of constant friction coefficients inadequately represents the dynamic tribological states within tool–chip contact zones, which vary with machining parameters and localized interfacial conditions. Research [23,24] confirms that friction coefficients exhibit dependencies on relative slip velocity, interfacial temperature, and normal contact stress. To address this, the variable friction coefficient model developed by Zou et al. [25] is implemented, mathematically formulated as

(6) $$\left\{\begin{array}{c} \mu =ae^{-bv}\left[1-{\left(\frac{T}{T_{m}}\right)}^{c}\right]/\sigma_{n}^{d}\\ \tau =min\left(\mu \sigma_{n},\tau_{max}\right) \end{array}\right.$$

In the equation, T  represents the average temperature on the rake face; $T_{m}$ is the material melting point; $\sigma_{n}$ denotes the normal stress at the contact interface; v  is the relative slip velocity; and a,b,c and  d  are constants with values of 0.4, 0.00027, 4.3, and 0.15, respectively. $\tau_{max}$ is the critical shear stress, which depends on temperature and the friction coefficient, and its expression is as follows:

(7) $$\tau_{max}=\left\{\begin{array}{c} \left[0.497+0.507\left(\mu -0.3\right)\right]\times \left(955.3-0.943T\right),\mu <0.48\\ \frac{1}{\sqrt{3}}\left(955.3-0.943T\right),\mu \geq 0.48 \end{array}\right.$$

#### 2.2.3. Machining Finite Element Model

**Figure 2:**
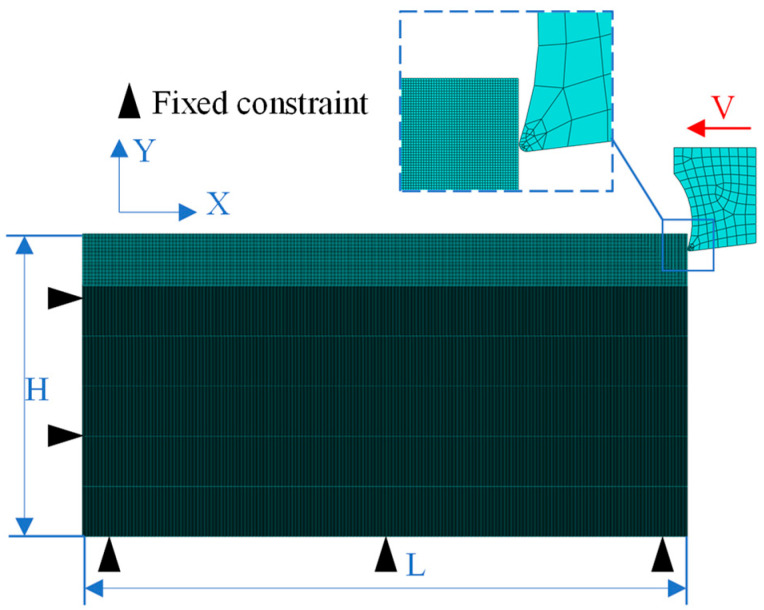
Simplified 2D finite element cutting model.

Constrained boundaries are imposed on the workpiece’s bottom and side surfaces, with the tool assigned an initial velocity. The workpiece model (L = 2 mm, H = 1 mm) incorporates a separation line positioned 0.6 mm from the machined surface, defining a 7 μm resolution mesh refinement zone between the line and the machined surface, while the remaining region adopts a coarse-mesh substrate. The tool and workpiece are discretized using a continuum plane element, 3-node thermal coupling (CPE3T) and continuum plane element 4-node reduced-integration thermal coupling (CPE4RT), respectively. The version of the ABAQUS software employed in this manuscript is 2020. As the software lacks built-in support for the TANH constitutive model and the variable friction coefficient model, it is necessary to develop subroutines and integrate them into the finite element model to make simulation possible. The feasibility of both models has been validated in Zou’s research [25,26]. Given the dominance of depth-of-cut over feed rate in metal cutting, the process is modeled as a two-dimensional orthogonal plane strain problem to balance accuracy and computational efficiency, avoiding three-dimensional (3D) simulation bottlenecks. The resulting 2D orthogonal turning finite element model is depicted in Figure 2.

### 2.3. Simulation-Based Cutting Performance Analysis of Tool Groove Geometries

**Figure 3:**
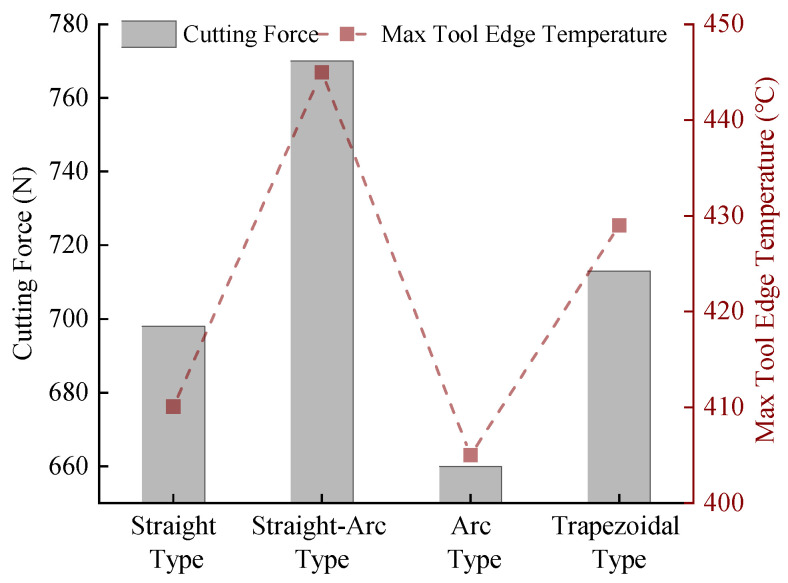
Variation in cutting performance with micro-groove structure.

**Figure 4:**
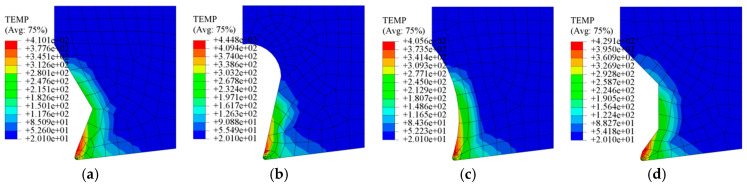
Temperature distribution of the tool tip with different groove structures: (**a**) Straight Type; (**b**) Straight-Arc Type; (**c**) Arc Type; and (**d**) Trapezoidal Type.

A comparative analysis of simulated cutting forces and maximum tooltip temperatures reveals distinct performance trends among the four micro-groove structures, as summarized in Figure 3. The Straight-Arc Type generates the highest cutting forces and peak tooltip temperatures, followed sequentially by the Trapezoidal Type, Straight Type, and Arc Type, which yield the most favorable thermomechanical outcomes. Figure 4 further illustrates temperature distribution patterns, showing that Trapezoidal and Straight-Type grooves produce larger high-temperature zones than the Arc and Straight-Arc Types. This disparity arises from abrupt geometric transitions in the rear sections of the two former structures, triggering secondary chip–groove collisions during chip flow. Conversely, the Arc Type’s smooth curvature ensures uniform temperature gradients without localized thermal spikes. These findings confirm that micro-groove geometry directly influences cutting temperatures and chip formation mechanisms under identical machining conditions.

**Figure 5:**
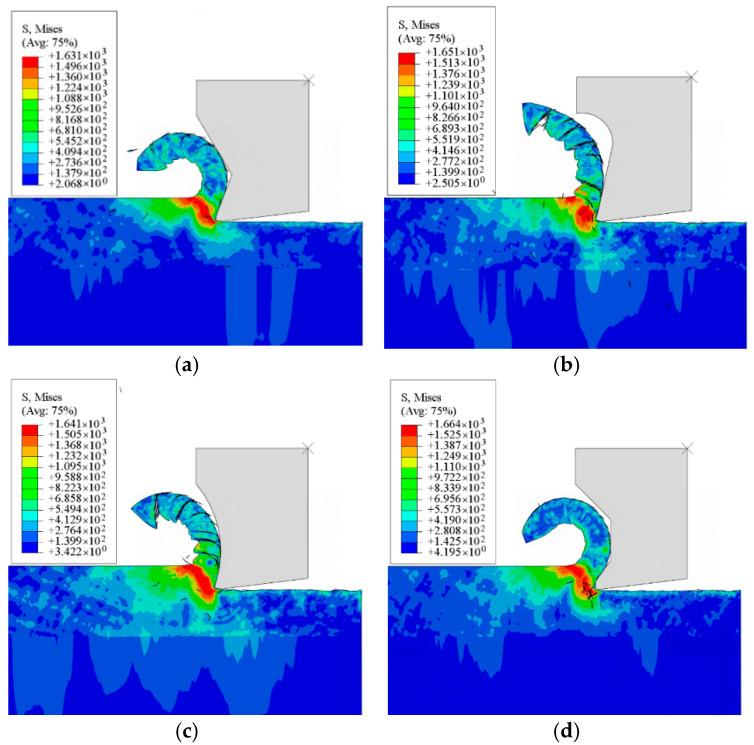
Four tool configurations: (**a**) Straight Type; (**b**) Straight-Arc Type; (**c**) Arc Type; and (**d**) Trapezoidal Type.

Variations in micro-groove geometries result in distinct chip morphologies that critically govern cutting performance, surface integrity, and thermal dynamics, making chip observation a vital diagnostic tool for process analysis. Under standardized conditions (cutting speed = 150 m/min, feed rate = 0.3 mm/rev), Figure 5 compares chips generated by four micro-groove structures. The Straight-Arc and Arc Types induce severe plastic deformation, forming serrated chips with localized fractures, while the Straight and Trapezoidal Types produce continuous ribbon-like chips with minimal deformation. Notably, the Straight-Arc Type yields chips with the largest curl radii (lowest curling intensity), contrasting with the Arc Type, which achieves smaller radii and superior chip fragmentation due to enhanced stress concentration. Thermal analysis further reveals that the Arc Type exhibits smoother temperature gradients and significantly lower peak temperatures than the other configurations, coupled with optimal chip control. These findings validate the Arc Type’s superior performance, justifying its selection for subsequent structural optimization.

## 3. Iterative Multi-Objective Optimization Framework for Tool Structural Design

### 3.1. Development of an Integrated Thermomechanical Simulation Platform

#### 3.1.1. Parametric Modeling of Tool Geometries

**Figure 6:**
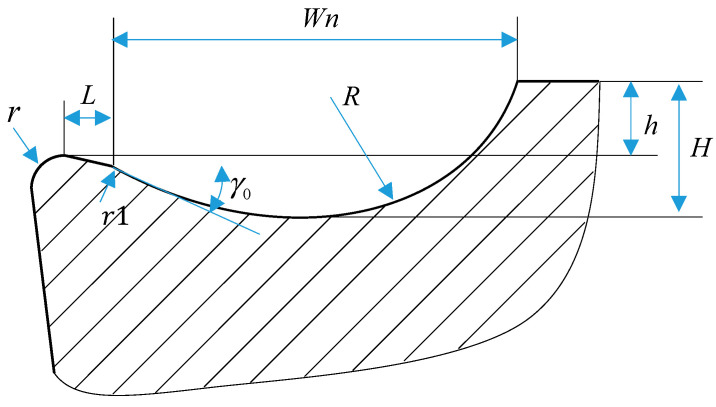
Schematic cross-section of circular micro-groove.

Defining the geometric parameters of arc-profile micro-grooves forms the foundation of parametric modeling. As illustrated in Figure 6, the tool cross-section integrates seven critical dimensions: tool nose radius, transition length (L), filet radius (r1), rake angle, edge height (h), groove depth (H), and groove width ${(W}_{n})$. To align with manufacturing feasibility, the tool nose radius is fixed at 0.02 mm, and r1 is maintained at 0.05 mm.

The width and depth of micro-grooves critically govern chip fragmentation and deformation dynamics during cutting. With Straight or Straight-Arc-Type grooves, increased groove width enlarges chip curl radii, making narrow grooves ideal for precision finishing and wider grooves suitable for roughing. Conversely, deeper grooves amplify the effective rake angle, suppressing chip deformation but degrading breakability. The groove’s width-to-depth ratio further modulates chip flow; high ratios drive geometric transitions from linear to arc-dominated profiles, minimizing groove depth while optimizing stress distribution. These transitions are quantified through the groove arc radius and depth in Equations (8) and (9), establishing a systematic framework for balancing chip control and machining efficiency.

(8) $$R=\frac{\sqrt{W_{n}^{2}+h^{2}}}{2sin\left(\gamma_{0}+arctan\left(h/W_{n}\right)\right)}$$

(9) $$H=h+\left(1-sin\gamma_{0}\right)R$$

At low width-to-depth ratios, the micro-groove geometry evolves from an Arc-Type to a Straight-Arc-Type configuration. Under these conditions, the groove depth attains its maximum value, while the corresponding arc radius is defined by Equation (10).

(10) $$R=\frac{w_{n}+h\cot\gamma_{0}}{1+tan\frac{\gamma_{0}}{2}+cot\gamma_{0}}$$

The parametric modeling process for the cutting tool uses the transition segment’s starting point as the reference to establish a Cartesian coordinate system, with  L, $W_{n}$, and H defined as dependent variables. Adjusting these variables governs the tool’s geometric configuration. Combining extensive cutting simulations with physical dimensional constraints, the permissible value ranges for these optimization parameters are empirically derived and tabulated in Table 5.

#### 3.1.2. Multi-Objective Optimization Criteria

Metal cutting represents a complex interplay of material removal, chip generation, and progressive tool wear. Chip morphology and dynamics critically modulate cutting forces, thermal gradients, and wear mechanisms, ultimately governing workpiece surface quality. Conventional tool optimization strategies, constrained by single-objective frameworks, struggle to balance competing performance metrics. This study advances a tri-objective paradigm targeting minimized cutting forces, thermal dissipation, and wear rates. The Usui wear model—an enhanced iteration of the Archard framework integrating temperature, contact stress, and slip velocity dependencies—quantifies wear progression. Empirical validation confirms its reliability, with governing equations formalized in Equation (11).

(11) $$\frac{dW}{dt}=apVe^{-b/T}$$

**Figure 7:**
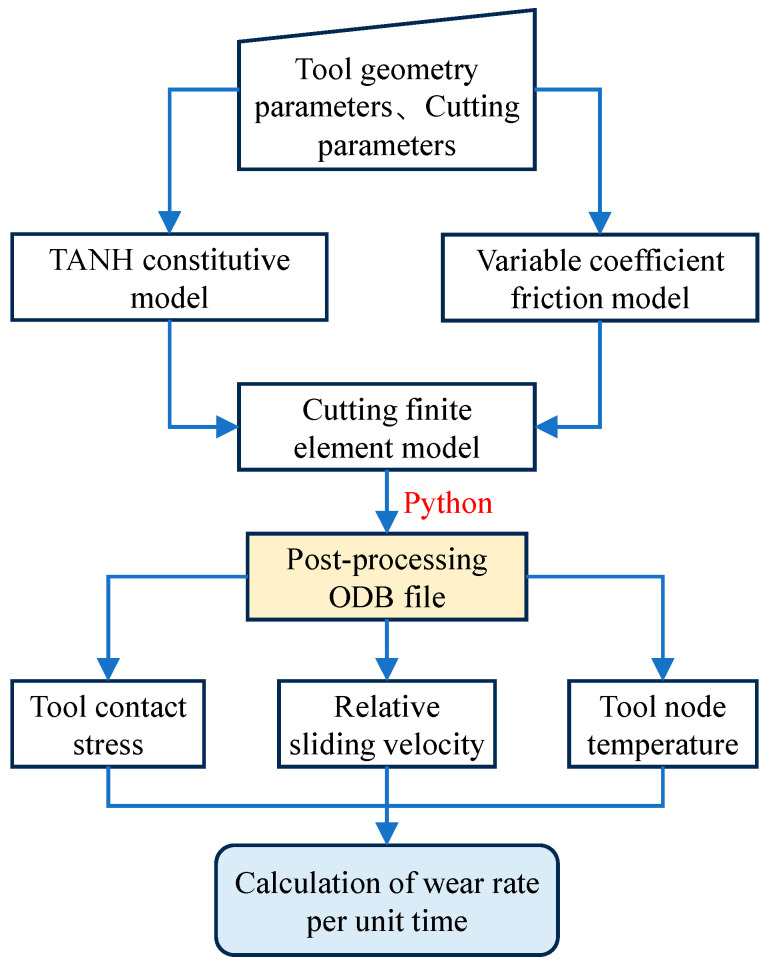
Process for calculating tool wear rate.

The equation defines p as contact normal stress, V as relative slip velocity, T as tool nodal temperature, and  a and b  as empirical coefficients (a = 10^−7^*,* b = 855). While cutting forces and temperatures are directly retrievable from ABAQUS post-processing, tool wear rates necessitate custom computational workflows. The simulation framework, already incorporating co-developed subroutines for the TANH constitutive model and variable friction model, adopts a discrete wear progression hypothesis: total cutting time is partitioned into uniform intervals  ∆t, each with constant wear rates. Incremental wear per interval equals  ∆t × wear rate. To mitigate subroutine variable conflicts that destabilize convergence, a Python-based pipeline extracts contact stresses, slip velocities, and nodal temperatures from stable cutting increments in ODB files. These data drive wear rate calculations for time-resolved wear quantification, as detailed in Figure 7.

For multi-objective optimization problems, two primary approaches are generally employed. The first involves converting the multi-objective problem into a single-objective one through linear weighting. However, this method suffers from the drawback of dimensional heterogeneity among objectives, making weight determination highly subjective. Additionally, while single-objective optimization yields a globally optimal solution, multi-objective optimization produces a solution set where individual solutions may excel in certain objectives but underperform in others. This implies that no single solution within the set is universally optimal, a phenomenon termed the Pareto optimal solution set (or non-dominated solution set). To address this, the present study employs the NSGA-II with elitist strategies to derive the Pareto front for three cutting performance metrics. The NSGA-II parameters are configured with a population size of 50, crossover probability of 0.7, and maximum iterations of 800. The mathematical formulation of the multi-objective optimization framework is as follows:

(12) $$\begin{array}{c} Find:\left(L,W_{n},H\right)\\ Min:E=\left(minF,minT_{max},minW\right)\\ subject\;to:\left\{\begin{array}{c} L_{min}\leq L\leq L_{max}\\ {W_{n}}_{min}\leq W_{n}\leq {W_{n}}_{max}\\ H_{min}\leq H\leq H_{max} \end{array}\right. \end{array}$$

#### 3.1.3. Optimization Outcomes

**Figure 8:**
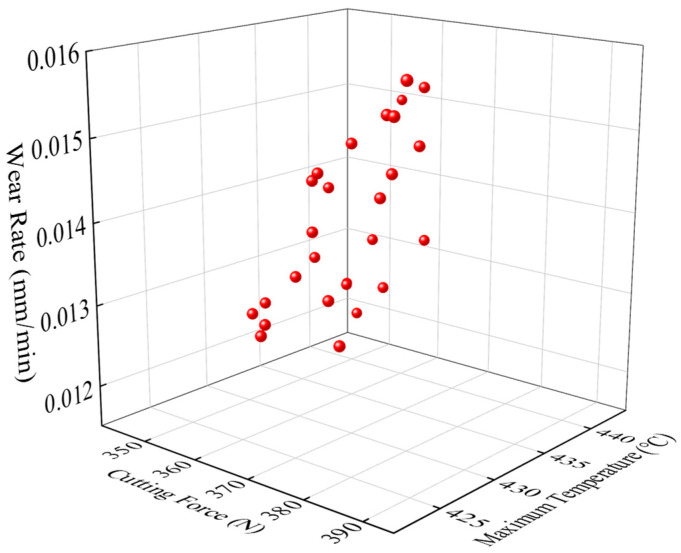
Pareto front of multi-objective optimization models.

Automated via Python 3.12.x scripting, the 2D turning FEM workflow executes parametric modeling, simulation, and extraction of cutting forces, temperatures, and wear rates. An NSGA-II-driven multi-objective framework inversely optimizes the micro-groove’s structural parameters, generating the Pareto front in Figure 8 and solution subsets in Table 6. Analytical results demonstrate a positive correlation between wear rate and cutting force/temperature, alongside an inverse force–temperature relationship. Integrated evaluation of all objectives prioritizes wear rate control (threshold: ≤0.013 mm/min) followed by thermal mitigation, identifying Solution Group 4 (L = 0.13 mm, $W_{n}$ = 1.78 mm, H = 0.45 mm) as the optimal trade-off configuration.

## 4. Performance Simulation and Manufacturing of Optimized Micro-Groove Tools

### 4.1. Cutting Performance Evaluation

**Figure 9:**
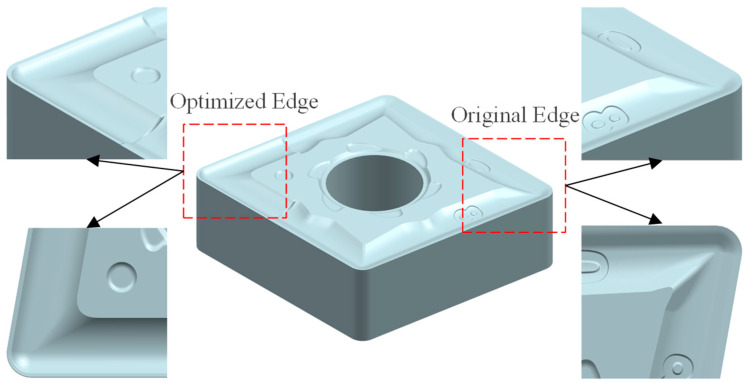
Three-dimensional structure of the tool.

The optimized tool’s cutting performance is validated by integrating its 2D micro-groove geometry into the original tool’s 3D model while preserving the nose profile. Coordinate transformations and surface reverse engineering techniques generate the final 3D optimized configuration. Figure 9 contrasts the edge topography of the original and optimized tools, highlighting geometric refinements from the micro-groove integration.

**Figure 10:**
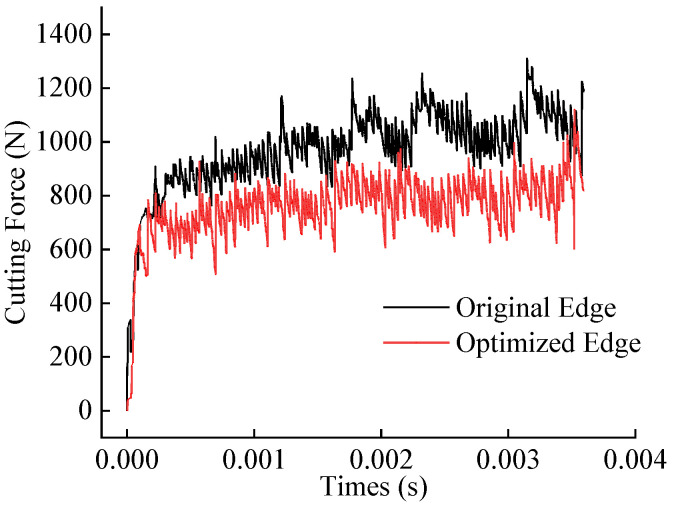
Comparison of simulated cutting forces between original and optimized tool.

**Figure 11:**
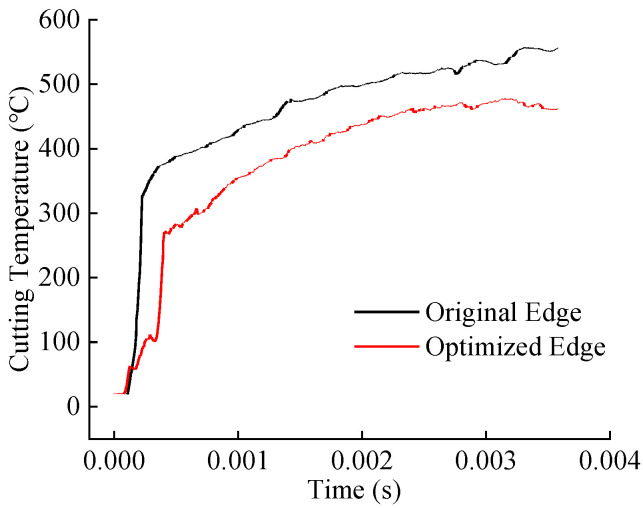
Comparison of simulated cutting temperatures between original and optimized tool.

Simulation studies were conducted under industry-recommended cutting parameters (cutting speed: 60 m/min, feed rate: 0.2 mm/rev, and depth of cut: 1.5 mm) to compare the cutting forces, temperatures, tool wear, and chip curling behavior between the original and optimized tools. Figure 10 and Figure 11 display the cutting force and temperature evolution over time for both tools. The optimized tool exhibits lower average cutting forces and temperatures compared to the original tool. Calculations based on steady-state cutting data reveal reductions of approximately 19.3% in cutting force and 14.2% in cutting temperature.

**Figure 12:**
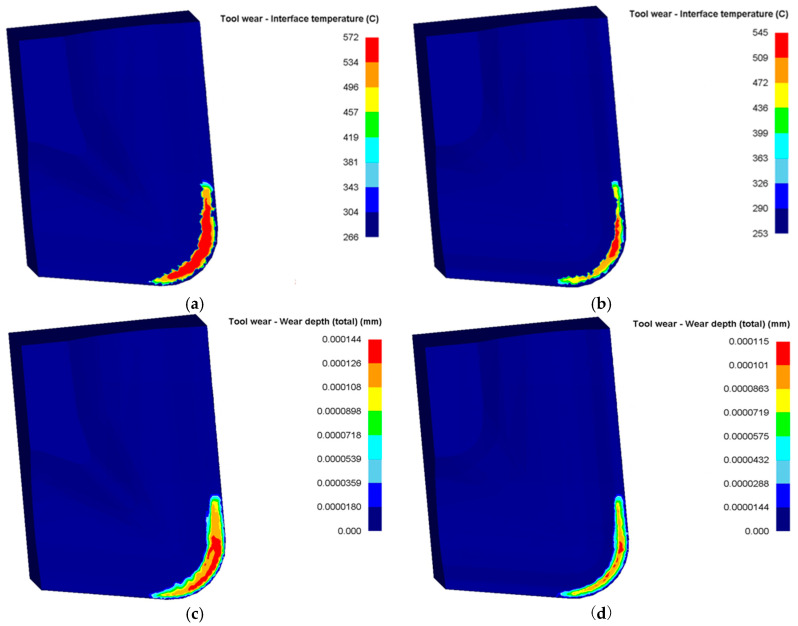
Distribution cloud of temperature and wear of original and optimized tool: (**a**) original edge temperature; (**b**) optimized edge temperature; (**c**) original edge wear depth; (**d**) optimized edge wear depth.

Figure 12 presents the temperature and wear distribution contour maps on the rake faces of the original and optimized tools. The results indicate that the original tool exhibits higher contact temperatures and wear magnitudes on its rake face compared to the optimized tool. Specifically, the original tool’s maximum rake face temperature exceeds that of the optimized tool by 30 °C. At equivalent cutting durations, the cumulative wear depth of the original tool is 0.000144 mm, whereas the optimized tool achieves 0.000115 mm, a 20.1% reduction in wear depth. Furthermore, the optimized tool demonstrates smaller high-temperature zones and wear distribution areas. These findings confirm that under identical cutting conditions, the optimized tool generates lower cutting temperatures and forces, reduces thermomechanical loads on the rake face, and enhances wear resistance.

**Figure 13:**
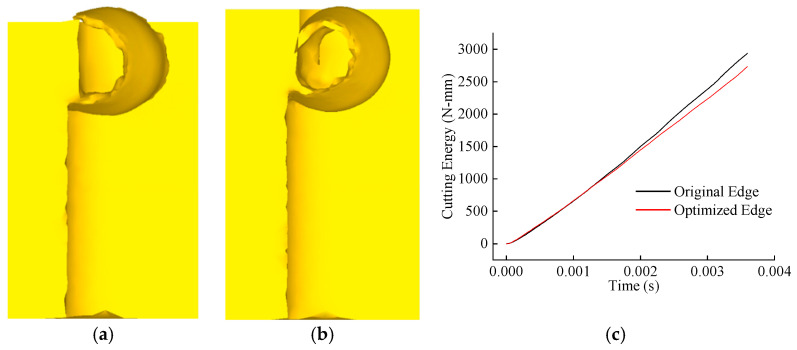
Comparison of chip morphology and cutting energy: (**a**) original edge chip; (**b**) optimized edge chip; (**c**) comparison of cutting energy.

Under identical cutting durations, the chip morphologies of the original and optimized tools are compared, and the energy consumption curves during cutting are extracted, as shown in Figure 13. The results reveal that while chip morphologies between the two tools are similar, the optimized tool produces chips with smaller curl radii and reduced thickness. Additionally, the optimized tool exhibits lower energy consumption during cutting compared to the original tool. This indicates that the optimized tool achieves superior chip-breaking efficiency and optimizes the distribution of mechanical–thermal–stress loads during the cutting process.

### 4.2. Structural Integrity Verification

**Figure 14:**
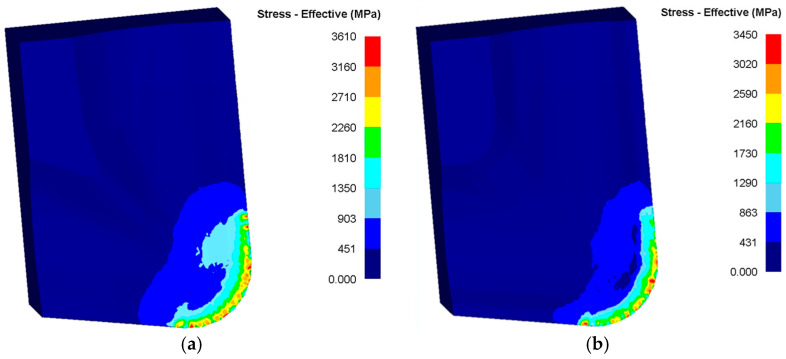
Tool stress distribution: (**a**) original tool; (**b**) optimized tool.

Conventional finite element models assign the tool as a rigid body, omitting strength effects and precluding edge failure prediction. To ensure structural credibility of the optimized tool pre-fabrication, an elastic model interpolates steady-state force fields onto the tool surface, resolving stress distributions (Figure 14). Both tools exhibit tooltip stress concentrations, yet the original design shows broader high-stress zones, confirming that the optimized geometry reduces tool–chip contact area and localized thermomechanical loads. Benchmarking against cemented carbide’s compressive strength (4.6 GPa), the optimized tool’s maximum equivalent stress (3.2 GPa) remains within safe limits, validating operational reliability under machining loads.

### 4.3. Tool Solid Preparation

**Figure 15:**
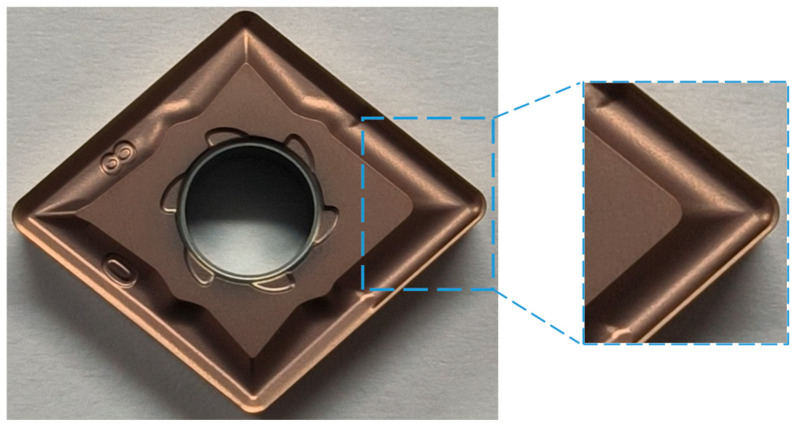
Solid model of optimized tool.

The optimized tool was manufactured based on the original tool via powder metallurgy compaction, with its surface subsequently treated by Physical Vapor Deposition (PVD). It features a substrate of fine-grained WC-Co carbide, suitable for machining titanium alloys, and a TiAlN coating approximately 3 μm thick. Owing to commercial confidentiality, the precise substrate composition, coating chemical formula, and deposition parameters are not disclosed. All tools used for comparison, however, were produced with identical materials and manufacturing processes to ensure that any performance differences are solely attributable to optimizations in the geometric design. Figure 15 showcases the finalized tool, demonstrating successful integration of structural and surface modifications.

## 5. Experimental Investigation of Optimized Tool Cutting Performance

### 5.1. Cutting Force Benchmarking

**Figure 16:**
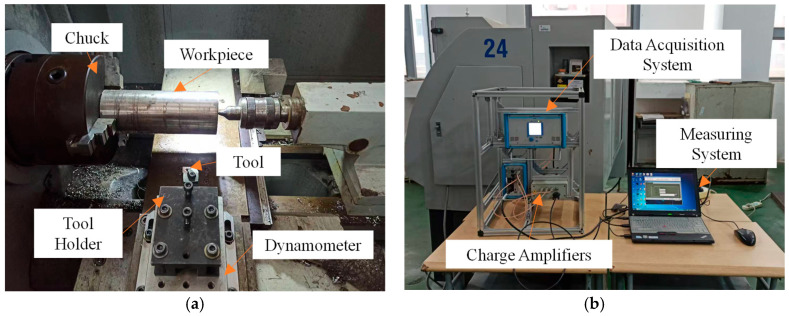
Cutting experiment site: (**a**) cutting experimental platform; (**b**) cutting force measurement system.

**Figure 17:**
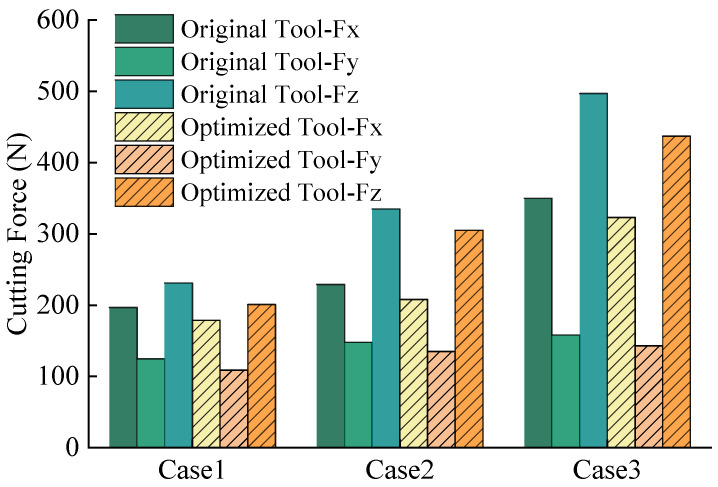
Comparison of cutting forces between original and optimized tools.

Post-fabrication validation tests evaluate the optimized tool’s performance on a C6136HK (manufacturer: Haishu machinery, Taian, China) computerized numerical control (CNC) lathe with an 80 mm diameter Ti6Al4V workpiece (Figure 16). Under identical parameters (Table 7), triplicate trials ensure statistical reliability, with averaged cutting forces during steady-state conditions plotted in Figure 17. The results demonstrate proportional force increases with feed per tooth and depth of cut. Under identical conditions, the optimized tool exhibits reduced principal cutting force, feed resistance, and depth-direction resistance compared to the original tool. The principal cutting force demonstrates the largest average reduction of 12.6%, confirming the optimized tool’s efficacy in lowering average cutting forces.

### 5.2. Chip Morphology Characterization

**Figure 18:**
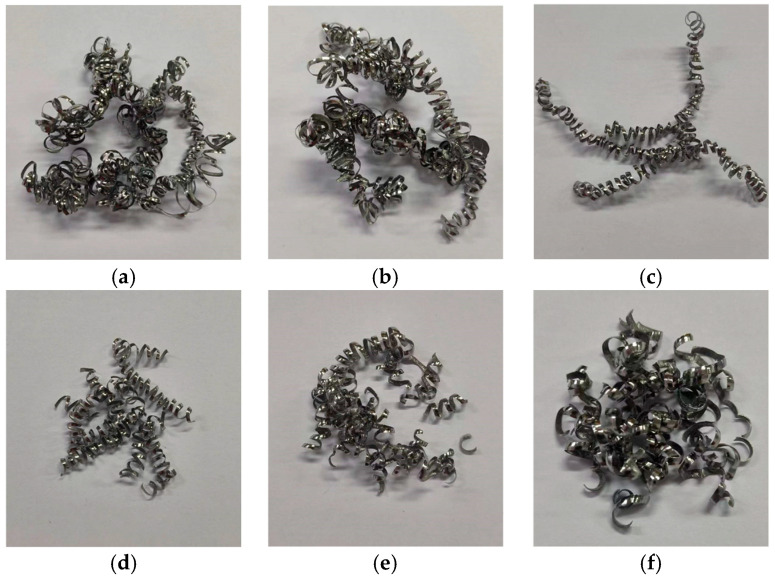
Comparison of chip morphology between original and optimized tool: (**a**) Case 1—Original Tool; (**b**) Case 2—Original Tool; (**c**) Case 3—Original Tool; (**d**) Case 1—Optimized Tool; (**e**) Case 2—Optimized Tool; and (**f**) Case 3—Optimized Tool.

Chips from all experimental groups in Table 7 were collected, as shown in Figure 18. Under the three cutting parameters, both the original and optimized tools produce spiral-shaped chips. The original tool generates longer, more continuous chips with lower fracture frequency, whereas the optimized tool exhibits significantly higher chip breakage rates. Metallographic specimens are prepared via hot mounting, and chip morphologies are examined using an S-EYE industrial camera microscope (manufacturer: Hayear, Shenzhen, China). The degree of chip serration is quantitatively characterized through geometric parameters [27]

(13) $$G_{s}=\frac{H-h}{H}$$

**Figure 19:**
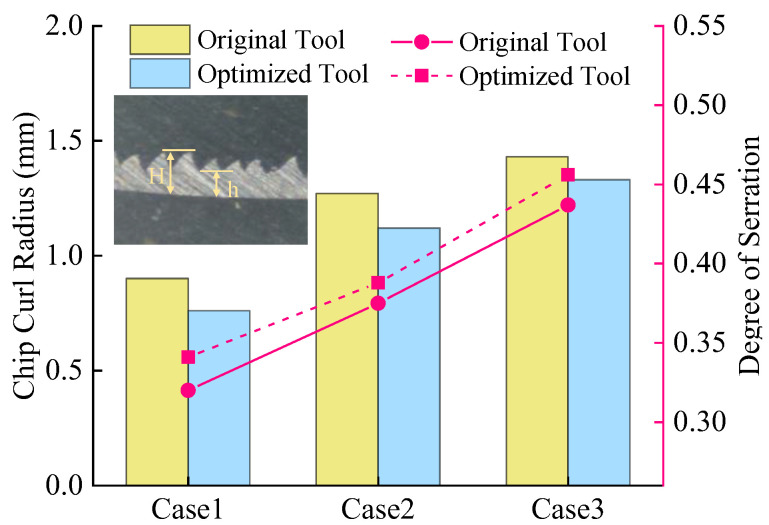
Comparison of chip profile data between original and optimized tool.

In the equation, $G_{s}$ represents the degree of serration. The distance between the serrated chip crest and the chip base layer is defined as the crest height H, while the distance between the serrated chip valley and the chip base layer is termed the valley height h. A uniform segment of the chip profile is selected, and three measurements of crest height and valley height are taken to calculate the average values. Chip curl radii and serration degrees are measured for both tools, as illustrated in Figure 19. The results demonstrate that increasing feed rate enlarges chip curl radii and intensifies serration. This is attributed to higher cutting forces and temperatures at elevated feed rates, which amplify plastic deformation and frictional interactions in the cutting zone, promoting chip bending and serrated chip formation. Quantitative analysis reveals that the optimized tool reduces average chip curl radius by 11.4% compared to the original tool but increases serration degree by 4.5%, validating its superior chip-breaking capability.

### 5.3. Tool Life Assessment and Wear Mechanism Characterization

**Figure 20:**
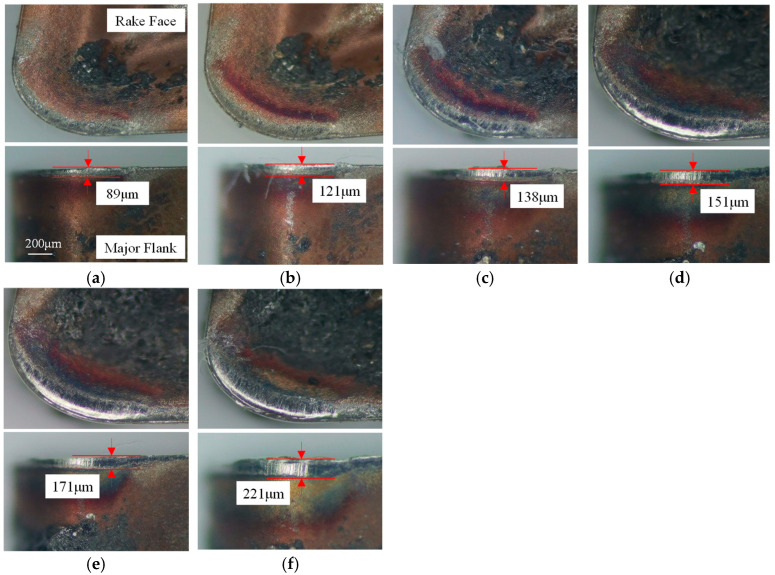
The evolution of wear of the original tool: (**a**) 2 min; (**b**) 4 min; (**c**) 6 min; (**d**) 8 min; (**e**) 10 min; and (**f**) 12 min.

**Figure 21:**
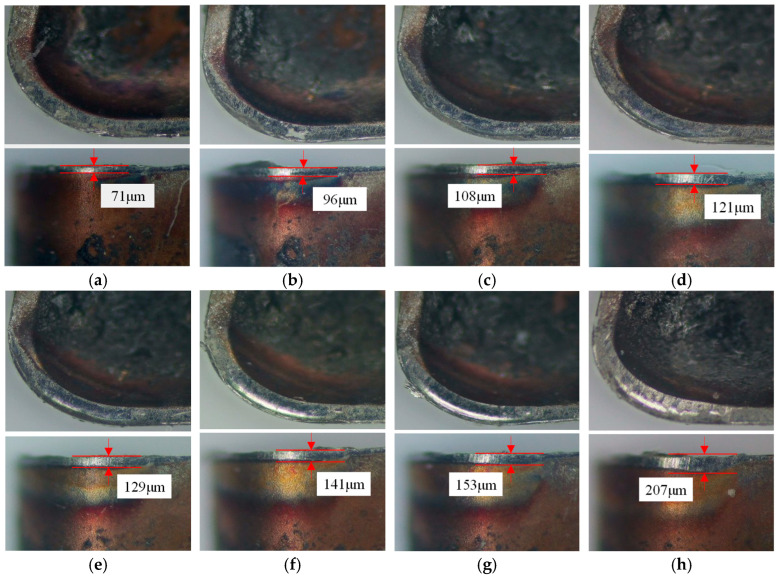
The evolution of wear of the optimized tool: (**a**) 2 min; (**b**) 4 min; (**c**) 6 min; (**d**) 8 min; (**e**) 10 min; (**f**) 12 min; (**g**) 14 min; and (**h**) 18 min.

Tool wear, driven by thermomechanical loads during cutting, compromises tool durability and machining stability, ultimately degrading surface quality. To rigorously assess the optimized tool, rake and flank wear evolution are analyzed under fixed parameters (cutting speed = 60 m/min, depth of cut = 1 mm, and feed rate = 0.10 mm/rev). Operators dismount the tool every two minutes for depth-of-field microscopy at the same scale, measuring flank wear land width until reaching the 200 μm failure threshold. Figure 20 and Figure 21 contrast wear morphologies, revealing that the optimized tool delays flank wear propagation and minimizes rake face abrasion compared to the original design, aligning with its enhanced thermal stress resistance.

**Figure 22:**
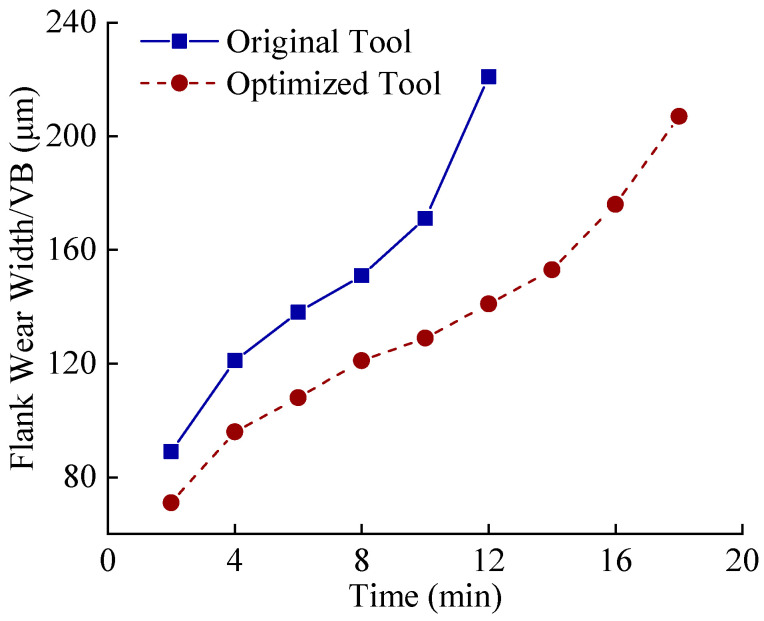
Curve of average flank wear with time.

During initial wear, intense friction strips the rake face coating. At 6 min, the original tool loses nearly all coating in the tool–chip contact zone, exposing the substrate, while the optimized tool retains partial coating integrity with superficial abrasion. By 12 min, the original tool’s flank wear land width reaches 221 μm, indicating it has exceeded the predefined wear threshold (200 μm) after 10 min. In contrast, the optimized tool remains in the stable wear phase with a flank wear land width of 141 μm at 12 min, only reaching the threshold (207 μm) at 18 min. This represents a 33.3% improvement in tool life for the optimized tool. Additionally, the original tool exhibits significantly larger rake face wear areas compared to the optimized design, attributed to the latter’s innovative geometry reducing tool–chip contact length and thermomechanical loading. By measuring the flank wear land widths of the original and optimized tools at identical time intervals, as shown in Figure 22, the growth rate of flank wear land width over cutting time reveals that both tools are in the initial wear stage within the first 4 min. The original tool transitions to the stable wear stage between 4 and 8 min, while the optimized tool maintains stable wear from 4 to 14 min. Post stable wear, wear rates accelerate until reaching the wear threshold. During this phase, the original tool’s flank wear land width increases from 171 μm to 221 μm (Δ50 μm), accompanied by micro-chipping, whereas the optimized tool exhibits a smaller increase of Δ31 μm while retaining intact edge topography. This indicates superior wear resistance and tooltip strength in the optimized tool during the late stable wear phase until threshold attainment.

**Figure 23:**
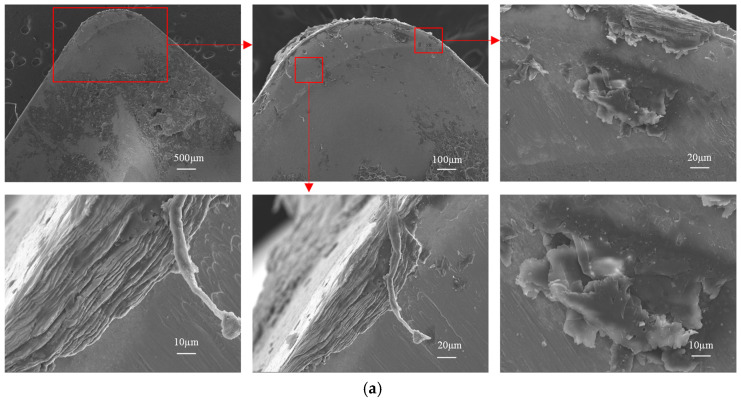

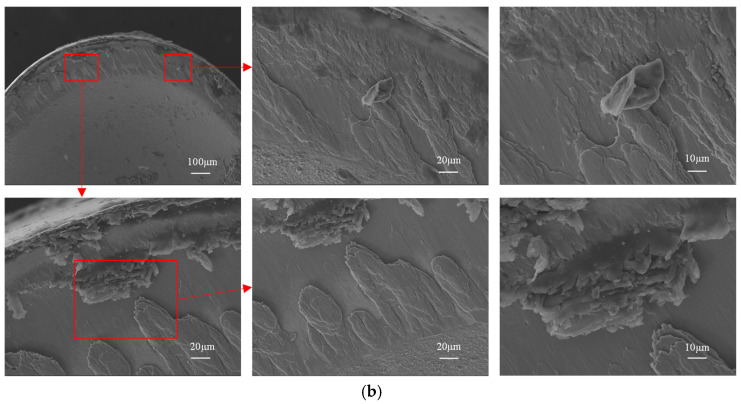
SEM morphology of the rank wear: (**a**) original tool -rake face; (**b**) optimized tool rake face.

The low thermal conductivity of Ti6Al4V elevates temperatures in the tool–chip contact zone, and exhibits strong chemical affinity with the cobalt (Co) binder phase in cemented carbide tools, rendering it highly susceptible to interdiffusion and material adhesion. As a result, adhesive wear constitutes the predominant mechanism for tool wear and failure. Figure 23 contrasts rake face scanning electron microscope (SEM) morphologies by Regulus8100 (manufacturer: Hitachi, Tokyo Metropolis, Japan), wherein Figure 23a displays the rake face wear morphology of the original tool, while Figure 23b shows that of the optimized tool. The original tool reveals dense adhered deposits (formed under thermomechanical loads), whereas the optimized tool exhibits sparser adhesion due to minimized contact area and suppressed cutting forces/temperatures. The original tool further suffers from unstable adhesion near the cutting edge, manifesting as cyclic adhesion buildup and spalling. These fluctuations generate localized stress concentrations, degrading surface finish and accelerating tool degradation. In contrast, the optimized geometry stabilizes interfacial interactions, mitigating adhesion-driven instability.

**Figure 24:**
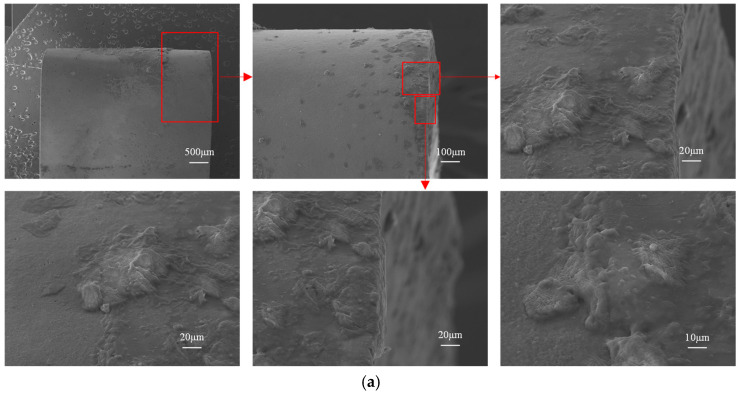

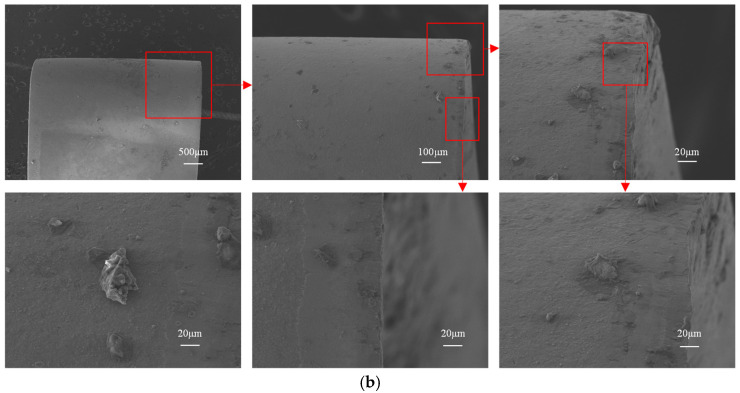
SEM morphology of the flank wear: (**a**) original tool flank face; (**b**) optimized tool flank face.

Figure 24 contrasts the flank wear SEM morphologies of both tools, wherein Figure 24a displays the flank wear morphology of the original tool, while Figure 24b shows that of the optimized tool. The original tool’s flank face reveals dense adhered deposits and irregular scratches within the wear land, coupled with severe micro-chipping along the cutting edge. In contrast, the optimized tool demonstrates sparser adhesion, smoother wear patterns, and a geometrically stable edge. These differences stem from the optimized design’s ability to homogenize thermomechanical stresses and suppress interfacial instabilities, reducing adhesive wear and preventing sudden wear rate fluctuations. The resultant load uniformity enhances cutting-edge durability and process consistency, validating the structural efficacy of the micro-groove optimization.

**Figure 25:**
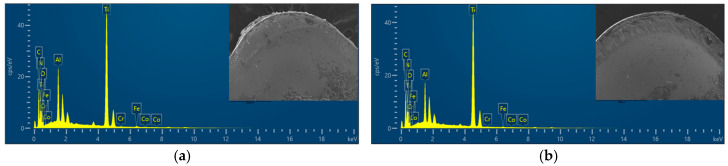
Energy spectrum of the rank face: (**a**) original tool rake face; (**b**) optimized tool rake face.

**Figure 26:**
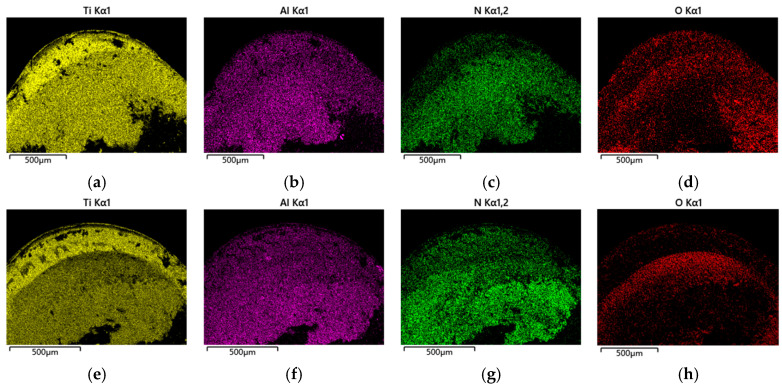
EDS element distribution: (**a**) Ti—Original Tool; (**b**) Al—Original Tool; (**c**) Co—Original Tool; (**d**) O—Original Tool; (**e**) Ti—Optimized Tool; (**f**) Al—Optimized Tool; (**g**) Co—Optimized Tool; and (**h**) O—Optimized Tool.

Figure 25 and Figure 26 present energy dispersive spectroscopy (EDS) analysis of rake face oxidative wear during high-speed turning. Elemental mapping reveals marginally higher Ti/Al concentrations on the original tool’s surface (attributed to workpiece material adhesion), whereas the optimized tool shows elevated nitrogen (N) content—a signature of retained coating material. This disparity confirms that the optimized design mitigates adhesion and preserves coating integrity under thermal cycling, with only partial delamination observed. In contrast, the original tool experiences extensive coating spallation, leading to pronounced substrate exposure. These findings validate the optimized geometry’s ability to resist oxidative degradation and prolong coating functionality in extreme machining conditions. The oxygen (O) content percentage serves as a critical indicator for assessing oxidative wear severity [28], the original tool’s rake face shows denser oxygen enrichment, particularly near the cutting edge where air infiltration intensifies. In contrast, adhesive friction suppresses oxygen diffusion in the tool–chip contact zone farther from the tooltip. The optimized tool’s micro-grooves exhibit 12% lower oxygen content than the original design, correlating with its reduced cutting forces and temperatures. These results indicate that the optimized tool improves thermomechanical load distribution near the tooltip under high-temperature and high-pressure conditions, reduces cutting temperatures, and significantly alleviates adhesive and oxidative wear.

### 5.4. Machined Surface Roughness Comparative Evaluation

**Figure 27:**
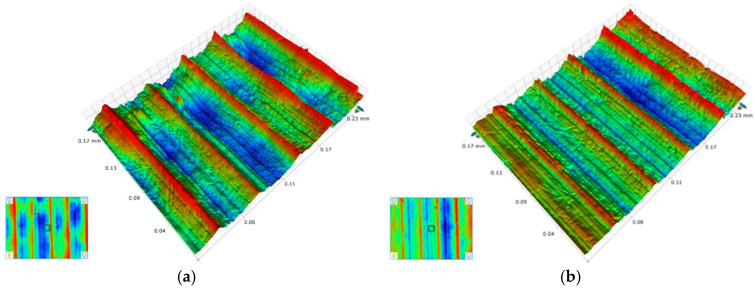

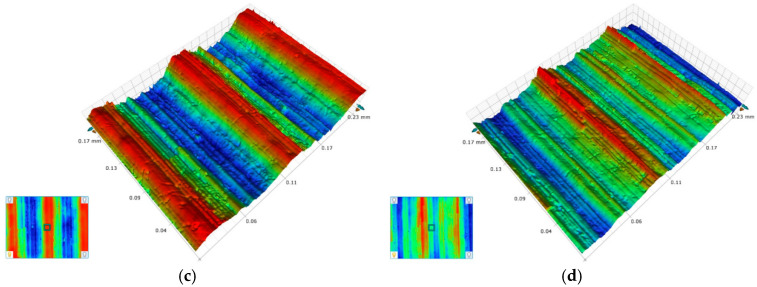
Surface morphology comparison: (**a**) f = 0.05 mm/rev—Original Tool; (**b**) f = 0.05 mm/rev—Optimized Tool; (**c**) f = 0.10 mm/rev—Original Tool; and (**d**) f = 0.10 mm/rev—Optimized Tool.

**Figure 28:**
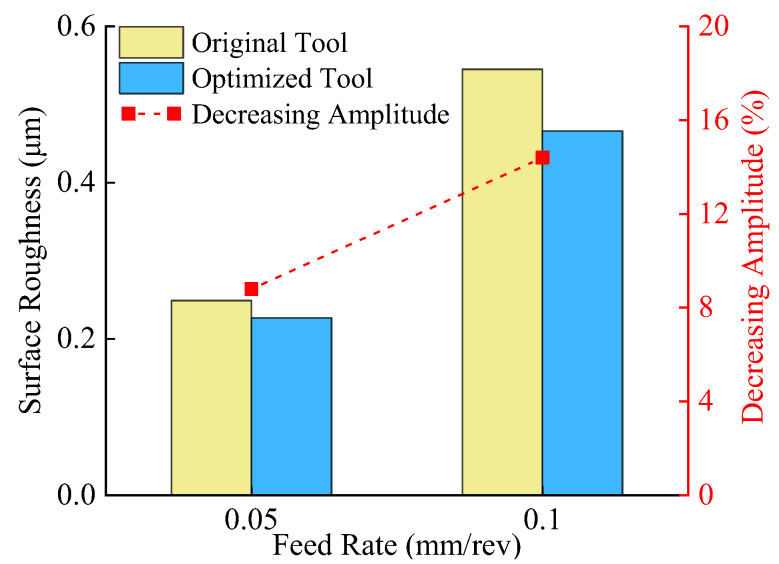
Comparison of surface roughness.

Surface roughness, a critical metric for evaluating surface quality, directly correlates with the wear resistance and fatigue resistance of machined surfaces. Figure 27 presents 3D surface microscopic topography of workpieces machined by the original and optimized tools under identical parameters (cutting speed = 60 m/min, depth of cut = 1 mm, feed rates = 0.05 mm/rev and 0.10 mm/rev). Both tools generate similar groove patterns on machined surfaces, but the original tool produces surfaces with larger peak-to-valley height variations and irregular scratches within valleys. In contrast, the optimized tool yields smoother peak ridges. Quantitative surface roughness analysis (Figure 28) confirms that the optimized tool reduces roughness compared to the original design, with greater reductions observed at higher feed rates. These results demonstrate that the optimized tool geometry reduces cutting forces and thermomechanical loads, enhancing machining stability and surface finish.

In summary, the optimized tool outperforms the original design in cutting force reduction, tool life extension, and chip-breaking efficiency, validating the feasibility of the multi-objective iterative optimization methodology for micro-groove structural design.

## 6. Conclusions

This manuscript develops a novel cutting tool design methodology based on the integration of co-simulation and intelligent optimization algorithms. It has been successfully implemented in the edge shape design of Ti6Al4V turning tools, demonstrating superiority over conventional methods in automation capability and physical reliability. The main conclusions are as follows:

(1) Under identical cutting parameters, the arc-type micro-groove structure improves cutting temperature distribution, reduces cutting forces, achieves smoother temperature variations, and significantly lowers the maximum tooltip temperature compared to other groove types, while demonstrating superior chip-breaking efficiency.
(2) With “minimum cutting force, minimum cutting temperature, and minimum tool wear rate” as optimization objectives, the transition segment length L, micro-groove width $W_{n}$, and micro-groove depth H were selected as structural parameters. The NSGA-II intelligent algorithm was employed to compute the optimal Pareto solution set, yielding the best micro-groove parameters: L = 0.13 mm, $W_{n}$ = 1.78 mm, H = 0.45 mm.
(3) Under identical simulation conditions, the optimized tool outperforms the original design, achieving a 19.3% reduction in cutting force, 14.2% decrease in temperature, and 20.1% lower wear rate per unit time, while concurrently minimizing the chip curl radius by 11.4%. These metrics validate the micro-groove geometry’s efficacy in balancing thermomechanical loads and enhancing machining efficiency.
(4) Turning experiments revealed that the optimized tool generates lower three-directional cutting forces compared to the original tool, with the largest average reduction in principal cutting force (approximately 12.6%). The optimized tool also exhibits significantly higher chip fracture frequency and smaller chip curl radii than the original tool, demonstrating superior chip-breaking performance.
(5) The optimized tool exhibits smaller adhered deposits on both rake and flank faces, with smoother flank wear morphology. When reaching the same wear threshold, the optimized tool achieves approximately 33.3% longer service life compared to the original tool. Elemental spectroscopy analysis confirms reduced oxidative wear in the optimized tool.

Future work will expand beyond macro-scale simulations to develop a micro–meso–macro multiscale coupled simulation framework and unify multi-source heterogeneous data for broader multi-objective design exploration.

## Figures and Tables

**Table 1:** Chemical composition of the Ti6Al4V (wt.%) [15].

Al	V	Fe	N + O	Ti
4.83–6.85	2.31–4.2	0.17	0.19 (max.)	Balance

**Table 2:** Ti6Al4V material performance parameters [16].

Density (kg/m^3^)	Young’s Modulus (GPa)	Poisson’s Ratio	Thermal Conductivity (W/m/K)	Specific Heat (J/kg/K)	Melting Point (°C)
4500	112	0.34	7.6	611	1650

**Table 3:** TANH constitutive model coefficients of Ti6Al4V [18].

A (MPa)	B (MPa)	C	n	m	a	b	c	d	$T_{r}$ (K)	$T_{m}$ (K)
968	380	0.0197	0.421	0.577	1.6	0.4	6	1	298	1878

**Table 4:** J-C damage failure parameters of Ti6Al4V [22].

$D_{1}$	$D_{2}$	$D_{3}$	$D_{4}$	$D_{5}$
−0.09	0.25	−0.5	0.014	3.87

**Table 5:** Optimized value intervals for the variables.

Variables	L	$W_{n}$	H
Corridor	[0.05, 0.17]	[1.5, 2.0]	[0.2, 0.5]

**Table 6:** Partial Pareto solution set of multi-objective optimization model.

Case	L (mm)	$W_{n}$ (mm)	H (mm)	F (N)	$T_{max}$ (℃)	W (mm/min)
1	0.11	1.65	0.39	346.3	442	0.013
2	0.16	1.93	0.43	352.5	434	0.014
3	0.12	1.70	0.47	367.6	425	0.015
4	0.13	1.78	0.45	364.4	423.2	0.013
5	0.15	1.84	0.43	361.7	432	0.014
6	0.12	1.69	0.42	371.9	435.7	0.013

**Table 7:** Cutting experiment program.

Case	Cutting Speed (m/min)	Feed Rate (mm/rev)	Cutting Width (mm)
1	60	0.05	1.0
2	60	0.10	1.5
3	60	0.15	2.0

## Data Availability

The original contributions presented in this study are included in the article. Further inquiries can be directed to the corresponding author.

## Abbreviations

TANH	Hyperbolic tangent
NSGA-II	Non-dominated sorting genetic algorithm II
FEM	Finite element method
2D	Two-dimensional
3D	Three-dimensional
J-C	Johnson–Cook
CPE3T	Continuum plane element, 3-node, thermal coupling
CPE4RT	Continuum plane element, 4-node, reduced integration, thermal coupling
PVD	Physical vapor deposition
CNC	Computerized numerical control
Co	Cobalt
SEM	Scanning electron microscope
N	Nitrogen
O	Oxygen
EDS	Energy-dispersive spectroscopy
